# Rethinking race-based interpretation in pediatric densitometry: a scoping review

**DOI:** 10.1093/jbmrpl/ziag028

**Published:** 2026-03-12

**Authors:** Amira Ramadan, Zakora Moore, Usman A Ahmed, Nessa Tantivit, Dhriti Aiylam, Chloe Rotman, Alicia Pendleton, Carter R Petty, Mali DiMeo, Amanda Grice, Valerie L Ward, Christina M Jacobsen, Nora E Renthal

**Affiliations:** Department of Pediatrics, Division of Endocrinology, Boston Children’s Hospital and Harvard Medical School, Boston, MA 02115, United States; Department of Pediatrics, Division of Endocrinology, Boston Children’s Hospital and Harvard Medical School, Boston, MA 02115, United States; Department of Pediatrics, Division of Endocrinology, Boston Children’s Hospital and Harvard Medical School, Boston, MA 02115, United States; Department of Pediatrics, Division of Endocrinology, Boston Children’s Hospital and Harvard Medical School, Boston, MA 02115, United States; Department of Pediatrics, Division of Endocrinology, Boston Children’s Hospital and Harvard Medical School, Boston, MA 02115, United States; Medical Library, Boston Children’s Hospital, Boston, MA 02115, United States; Department of Pediatrics, Division of Endocrinology, Boston Children’s Hospital and Harvard Medical School, Boston, MA 02115, United States; Biostatistics and Research Design (BARD) Center, Boston Children’s Hospital, Boston, MA 02115, United States; Department of Pediatrics, Division of Endocrinology, Boston Children’s Hospital and Harvard Medical School, Boston, MA 02115, United States; Office of Health Equity and Inclusion, Sandra L. Fenwick Institute for Pediatric Health Equity and Inclusion, Department of Health Affairs, Boston Children’s Hospital and Harvard Medical School, Boston, MA 02115, United States; Office of Health Equity and Inclusion, Sandra L. Fenwick Institute for Pediatric Health Equity and Inclusion, Department of Health Affairs, Boston Children’s Hospital and Harvard Medical School, Boston, MA 02115, United States; Department of Radiology, Boston Children’s Hospital and Harvard Medical School, Boston, MA, 02115 United States; Department of Pediatrics, Division of Endocrinology, Boston Children’s Hospital and Harvard Medical School, Boston, MA 02115, United States; Department of Pediatrics, Division of Endocrinology, Boston Children’s Hospital and Harvard Medical School, Boston, MA 02115, United States

**Keywords:** pediatric bone density, DXA, BMD, race, ethnicity, pediatric reference data, normative curves, health disparities

## Abstract

As pediatric populations in the United States (US) become increasingly diverse, current practices for interpreting bone density using DXA in children warrant reevaluation. The International Society for Clinical Densitometry currently recommends adjusting pediatric bone density Z-scores by race, sex, and age. However, race-based adjustments risk reinforcing disparities and perpetuating systemic inequities in pediatric bone health assessment. We conducted a scoping review of studies examining racial and ethnic differences in BMD among healthy US children, identifying 3960 records across 4 databases, of which 54 met inclusion criteria. Across these studies, reporting of race and ethnicity was inconsistent: although nearly all relied on self- or parent-report, none provided explicit definitions, and only 13% confirmed concordance across grandparents. Fifty percent of studies reported statistically significant racial differences in BMD, yet most did so without comprehensive covariate adjustment. By contrast, studies that accounted for height, lean mass, and pubertal status frequently found that differences attenuated or disappeared. These findings underscore the need to critically reconsider race-based adjustments in pediatric DXA interpretation. Developing and validating race-neutral reference standards, with attention to structural determinants and biologically relevant measures, such as stature, body composition, and pubertal timing, is essential for achieving a more equitable and clinically meaningful assessment of pediatric bone health.

## Introduction

The use of race in clinical decision-making is undergoing intense scrutiny across medicine. Historically treated as a biological category, race is now widely recognized as a social construct. While genetic variation exists across human populations, socially defined racial categories do not reliably capture this variation and are not a proxy for underlying biology. This reappraisal has prompted major organizations to reconsider the clinical use of race. For example, the American Academy of Pediatrics has required that race be described as a social rather than biological construct and has urged the retirement of race-based clinical tools, including its urinary tract infection guideline for febrile infants and children.^1^ Systematic reviews of pediatric clinical practice guidelines further show that race has often been incorporated in ways that risk worsening inequities, prompting national organizations to advocate for race-conscious frameworks that emphasize social determinants of health rather than presumed biological differences.^2^^,^^3^ Recent guidance from the National Academies of Sciences, Engineering, and Medicine similarly emphasizes the need for careful, context-specific use of race and ethnicity in biomedical research and clinical tools, cautioning against conflating socially defined categories with biology while supporting rigorous, context-specific approaches when race is collected to study structural determinants of health.^4^

This broader reexamination is directly relevant to pediatric bone health, where racial differences in bone density have long shaped the interpretation of DXA. Numerous studies consistently report higher BMC and areal BMD (aBMD) in Black children and adults compared to White, Asian, and Hispanic peers with findings often cited as contributing to their comparatively lower fracture risk. Based on these observations, the International Society for Clinical Densitometry (ISCD) recommends calculating pediatric DXA BMD Z-scores using reference data stratified by age, sex, and race/ethnicity, effectively dichotomizing children as “Black” or “non-Black.”^5^ In light of growing consensus that race is a social construct and mounting concerns about inequity, the validity and utility of this race-based practice are called into question, highlighting the importance of critical reevaluation.

Our recent work has raised concerns regarding the operational challenges of race-based pediatric DXA interpretation, including the absence of standardized guidance for assigning racial categories and uncertainty in interpreting scans for children of mixed identities.^6^ While direct evidence of harm from the use of race in DXA is limited, recent data show that collecting race and ethnicity data can negatively affect patient experience. Studies emphasize that the collection of ethnicity data is not experienced neutrally and may generate distress or concern about misuse unless the rationale, anticipated benefits, and safeguards are clearly articulated.^7^ Patients with prior experience of discrimination are less comfortable providing race information and more likely to worry that such information could be used to discriminate.^8^ These gaps underscore the broader risks of incorporating race into clinical algorithms: reinforcing inequities, introducing bias into decision-making, and potentially undermining trust for some patients when race/ethnicity is collected or applied without clear rationale and safeguards. To address these concerns, we conducted a scoping review to critically examine the strength and limitations of the evidence supporting race-based adjustments in pediatric DXA interpretation. Specifically, this review evaluates how race has been defined and operationalized across studies, whether key biological and social determinants of bone density have been adequately addressed, and whether the evidence justifies continued reliance on race-based adjustments in pediatric DXA.

## Materials and methods

### Search strategy and data sources

A medical librarian conducted systematic searches of PubMed, Embase, Scopus, and Web of Science, from database inception through May 28, 2024. The search strategy followed the Preferred Reporting Items for Systematic Reviews and Meta-Analyses extension for Scoping Reviews (PRISMA-ScR) guidelines. Search terms combined descriptors of pediatric populations (eg, child, adolescent, and youth) with terms related to race and ethnicity, BMD, bone mineralization, and DXA (Table S1). Citations were screened in Covidence systematic review software. Gray literature and conference proceedings were not included. A protocol was developed a priori but was not formally registered.

### Eligibility criteria

The objective of this review was to evaluate how race has been incorporated into the development and application of pediatric DXA reference ranges. Eligible studies included healthy pediatric populations (≤19 yr) assessed with DXA that either: (1) examined racial or ethnic differences in BMD and/or (2) contributed to the creation or justification of race-based adjustments for pediatric DXA-derived BMD Z-scores. During screening, studies from both U.S. and non-U.S. settings were initially considered, but the final analysis was restricted to U.S. cohorts to ensure consistency of sociocultural and healthcare contexts.

**Figure 1:**
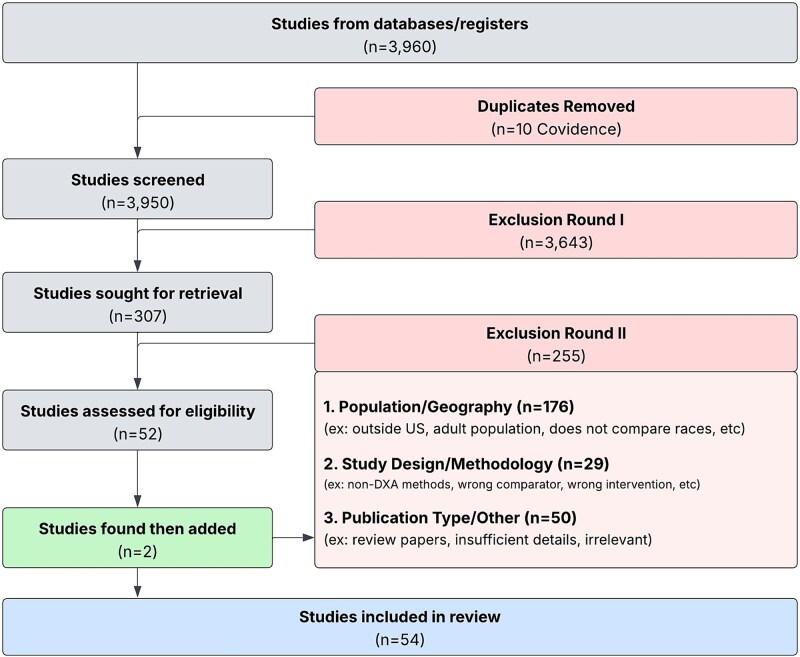
Screening and eligibility assessment for included studies. A total of 3960 studies were identified; 54 studies met inclusion criteria.

We excluded studies of children with chronic diseases or conditions known to affect bone metabolism, as the goal was to examine how race has been applied in interpreting DXA among otherwise healthy populations. Non-original reports (eg, editorials, narrative reviews, case series, or case reports), as well as non-English language publications, were also excluded. The study selection process is illustrated in a PRISMA-ScR flow diagram (Figure 1).

### Data charting process

A standardized data charting form was developed to systematically capture study characteristics and contextual variables. Extracted items included study design, DXA machine type, population demographics (age, race/ethnicity, pubertal stage, height, BMI, weight, body composition, and other biological factors), socioeconomic indicators, skeletal sites assessed, methods of race/ethnicity data collection, and primary findings related to racial or ethnic differences in BMD. The form was pilot tested on a subset of studies and refined iteratively to incorporate emerging themes specific to the operationalization of race in DXA research. Data extraction was conducted independently by 5 reviewers, with discrepancies adjudicated through discussion and consensus thereby ensuring both methodological rigor and interpretive alignment. Because included studies span several decades, racial terminology varied across original publications. For consistency, we standardized terminology for our analysis, using “Black” (in place of “African American”) and “White” (in place of “Caucasian”) throughout.

### Synthesis of results

Extracted data were summarized descriptively in tables and narratively synthesized. In line with scoping review methodology, no formal risk of bias or critical appraisal of study quality was performed. Findings were organized thematically according to whether studies reported racial or ethnic differences in BMD, and by their role in the development or application of race-based reference ranges.

## Results

### Study selection

Our initial search yielded 3960 papers, from which 10 duplicates were removed. After screening the remaining papers, 3643 were excluded, and the 307 that met the screening criteria were retrieved for further review. Additional screening led to the exclusion of more papers, and ultimately, 54 papers were selected for a full-text review, deemed eligible, and thus included in the full analysis as shown in Figure 1.

### Race and ethnicity reporting

Among the 54 included studies (Table 1), reporting of race and ethnicity was highly variable. Twelve studies (22%) did not specify how these data were obtained, while the majority, 42 studies (78%), relied on self- or parent-report via questionnaire. Of these, 7 studies (13%) imposed stricter criteria by requiring that all four grandparents of the child were of the same race.^9–15^ Despite frequent reliance on self-report, none of the studies provided explicit definitions of race or ethnicity, and methods of classification were rarely described in detail. In most cases, the approach to identifying race was not clearly detailed. A single recent study incorporated additional markers, including skin tone and genetic ancestry, highlighting emerging efforts to move beyond self-report race to provide a more nuanced understanding of racial and ethnic differences in BMD.^16^

**Table 1:** Studies by data source.

**Independent (34 studies)**			**BMDCS (12 studies)**			**NHANES (8 studies)**		
**Author**	**Year**	***N***	**Author**	**Year**	***N***	**Author**	**Year**	***N***
**Yanovski et al.**	1996	40	Kalkwarf et al.	2007	1554	Kelly et al.	2009	4582
**Moro et al.**	1996	375	Zemel et al.	2009	821	Looker et al.	2012	4406
**Morrison et al.**	1996	98	Short et al.	2011	1889	Looker et al.	2013	11 804
**Wang et al.**	1997	423	Zemel et al.	2011	2014	Fan et al.	2014	8056
**Ellis et al.**	1997	297	Lappe et al.	2015	1743	Suárez et al.	2017	8348
**Nelson & Simpson et al.**	1997	773	Short et al.	2015	2014	Duran et al.	2018	2026
**Nelson & Barondess et al.**	1997	734	McCormack et al.	2017	2014	Schafmeyer et al.	2022	6452
**Bachrach et al.**	1999	423	Kindler et al.	2019	2014	Schafmeyer et al.	2024	2398
**Barbeau et al.**	1999	71	Kindler et al.	2020	1554			
**Horlick et al.**	2000	336	Kalkwarf, Shepherd, & Fan et al.	2022	484			
**Yanovski et al.**	2000	118	Kalkwarf, Shepherd, & Hans et al.	2022	2014			
**Bray et al.**	2001	114	Zemel et al.	2025	2014			
**Wright et al.**	2002	31						
**Wong et al.**	2002	141						
**Tershakovec et al.**	2002	203						
**Henderson et al.**	2002	256						
**Hui et al.**	2003	232						
**Litaker et al.**	2003	219						
**Cromer et al.**	2004	422						
**Horlick et al.**	2004	1218						
**Afghani et al.**	2006	181						
**Ackerman et al.**	2006	926						
**Dorsey et al.**	2010	175						
**Hui et al.**	2010	188						
**Casazza et al.**	2010	270						
**Gutin et al.**	2011	660						
**Newton et al.**	2013	59						
**Cattran et al.**	2015	227						
**Hanks et al.**	2015	37						
**Misra et al.**	2017	60						
**Broadney et al.**	2018	594						
**Reyes et al.**	2020	72						
**Shypailo et al.**	2020	1079						
**Gordon et al.**	2024	557						

Studies are grouped according to the dataset from which participants were drawn. *Independent* refers to single-site or locally recruited cohorts not tied to large national or multicenter datasets. *BMDCS* indicates analyses based on the Bone Mineral Density in Childhood Study, a large multicenter U.S. cohort. *NHANES* refers to analyses using National Health and Nutrition Examination Survey data. Within each group, studies are ordered chronologically by publication year, and the total number of participants included in each analysis (*N*) is shown.

### Studies identifying racial/ethnic differences in bone density

Of the 54 studies included, 13 (24%) reported no meaningful racial or ethnic differences in BMD or related measures,^10^^,^^11^^,^^17–26^ 10 (19%) reported mixed or attenuated differences after covariate adjustment^9^^,^^14^^,^^15^^,^^27–33^; 27 (50%) reported statistically significant differences often without consideration of covariates,^12^^,^^13^^,^^16^^,^^34–57^ and 4 (7%) presented only descriptive data without statistical testing (Table 2 and Table S2).^58–61^ Distinct methodological patterns accounted for much of this variation. Studies reporting no racial differences more often used alternative outcomes like BMAD or evaluated bone accrual (rather than cross-sectional bone mass measures); however, use of BMAD did not uniformly eliminate racial differences across studies.^53^ Studies reporting no racial differences also applied more robust covariate adjustment (eg, height, weight, pubertal stage, and body fat distribution) or focused on populations where confounding was partly controlled by design, such as cohorts with obesity or studies of energy expenditure.

**Table 2:** Studies by reported racial/ethnic differences in pediatric BMD.

**No differences (*n* = 13)**					
**Author**	**Year**	***N***	**Author**	**Year**	***N***
**Morrison et al.**	1996	98	^*^Short et al.	2015	2014
**Barbeau et al.**	1999	71	^*^Lappe et al.	2015	1743
**Tershakovec et al.**	2002	203	^*^Cattran et al.	2015	227
**Afghani & Goran et al.**	2006	181	Broadney et al.	2018	594
**Dorsey et al.**	2010	175	Reyes et al.	2020	72
**Casazza et al.**	2010	270	^*^Kalkwarf, Shepherd, & Hans et al.	2022	2014
^*^Short et al.	2011	1889			
**Mixed/attenuated (*n* = 10)**					
**Moro et al. et al.**	1996	375	Hui et al.	2003	232
**Yanovski et al.**	1996	40	Ackerman et al.	2006	926
**Nelson & Simpson**	1997	773	Hui et al.	2010	188
**Yanovski et al.**	2000	118	#Duran et al.	2018	2026
**Wright et al.**	2002	31	^*^Zemel et al.	2025	2014
**Persistent differences (*n* = 27)**					
**Ellis et al.**	1997	297	#Looker et al.	2012	4406
**Nelson & Barondess et al.**	1997	734	Newton et al.	2013	59
**Bachrach et al.**	1999	423	#Looker et al.	2013	11 804
**Wang et al.**	1999	423	Hanks et al.	2015	37
**Horlick et al.**	2000	336	Misra et al.	2017	60
**Bray et al.**	2001	114	^*^McCormack et al.	2017	2014
**Henderson et al.**	2002	256	#Gállego Suárez et al.	2017	8348
**Wong et al.**	2002	141	^*^Kindler et al.	2019	2014
**Horlick et al.**	2004	1218	^*^Kindler et al.	2020	1554
**Cromer et al.**	2004	422	^*^Kalkwarf, Shepherd & Fan et al.	2022	484
^*^Kalkwarf et al.	2007	1554	#Schafmeyer et al.	2022	6452
^*^Zemel et al.	2009	821	Gordon et al.	2024	557
^*^Zemel et al.	2011	2014	#Schafmeyer et al.	2024	2398
**Gutin et al.**	2011	660			
**Descriptive (*n* = 4)**					
**Litaker et al.**	2003	219	#Fan et al.	2014	8056
**#Kelly et al.**	2009	4582	Shypailo & Wong et al.	2020	1079

Studies are grouped according to whether reported racial/ethnic differences were absent (“No differences”), attenuated with covariate adjustment (“mixed/attenuated”), persisted after adjustment (“persistent differences”), or were presented descriptively without statistical comparison (“descriptive”). Within each group, studies are ordered chronologically by publication year, and the total number of participants included in each analysis (*N*) is shown. ^*^indicates analyses based on the Bone Mineral Density in Childhood Study and # indicates analyses using National Health and Nutrition Examination Survey data.

^*^ BMDCS dataset, # NHANES dataset.

By contrast, studies reporting attenuated differences often analyzed BMC or aBMD at 1 or 2 skeletal sites with partial adjustment—most often with age, sex, height, weight, and sometimes Tanner stage—without consistently including lean mass, diet, activity, or socioeconomic status (SES). In these studies, apparent racial differences often diminished after anthropometric factor adjustment, and effects varied by skeletal site or pubertal timing.

Finally, studies reporting persistent differences were frequently large reference-curve or surveillance cohorts that emphasized BMC and aBMD, but adjusted only for age, sex, and height. These designs left residual Black vs non-Black differences across most skeletal sites without adjusting for puberty, lean mass, diet, activity, or SES. Reliance on older DXA methodologies and descriptive reference analyses could have also contributed to persistent findings. Taken together, these patterns indicate that the apparent magnitude of racial differences in pediatric bone density depends less on intrinsic biology than on the choice of metric, the rigor of covariate adjustment, and the analytic purpose of the study.

### Multiple comparisons testing

Interpretation of racial differences in BMD must account for the problem of multiple comparisons. As the number of statistical tests increases—across skeletal sites, age groups, and sex strata, the likelihood of detecting spurious association rises substantially. Among the 43 studies that reported statistically significant racial or ethnic differences in BMD, BMC, or related measures, most conducted numerous comparisons across skeletal sites (eg, TBLH, TB, and radius) and demographic subgroups yet often drew conclusions from only a subset of these results. Only 6 studies applied any statistical correction for multiple testing. In the absence of such correction, reported differences may reflect chance findings, potentially amplified by selective emphasis on findings that align with prior expectations.^14^^,^^23^^,^^24^^,^^33^^,^^37^^,^^51^

### Covariate analysis: mechanisms underpinning observed racial differences

Pediatric BMD is influenced by multiple biological, behavioral, and social factors. Adjustments for covariates, such as body size, lean and fat mass, pubertal status, physical activity, diet, and SES, have been shown to attenuate reported racial differences in BMD in adulthood^62^ and, to a lesser degree in 8 studies within this review.^12^^,^^22^^,^^27^^,^^28^^,^^30^^,^^31^^,^^33^^,^^36^ Across the 54 studies, however, statistical adjustment was highly inconsistent, and no study simultaneously incorporated all relevant covariates (Table 3). Critical determinants, such as pubertal stage and SES, were often omitted or only addressed through indirect or limited measures. These gaps in accounting for key determinants that impact bone accrual in the pediatric population underscore that apparent racial differences in pediatric BMD may reflect residual confounding rather than intrinsic biology, reinforcing the need for cautious interpretation and more comprehensive analytical approaches in future research.

**Table 3:** Covariates considered in pediatric BMD studies by race/ethnicity.

**Category**	***n***	**%**
**Race and ethnicity reporting**	**42**	**78**
**Provided definition of race**	**0**	**0**
**Specified method of race collection**	**42**	**78**
**Considered racial concordance of child with parents/grandparents**	**6**	**11**
**Considered the impact of puberty**	**39**	**72**
**Physical exam by study team**	**22**	**41**
**Limited study population to specific stage**	**6**	**11**
**Participant self-report**	**9**	**17**
**Mixed method/unclear**	**2**	**4**
**Considered the impact of body composition**	**35**	**65**
**Height-adjusted Z-scores**	**15**	**28**
**Weight/BMI**	**23**	**43**
**Lean/fat mass**	**18**	**33**
**Considered the impact of other biological variables**	**12**	**22**
**Nutrition**	**6**	**11**
**Physical activity**	**8**	**15**
**Birth control**	**1**	**2**
**Considered social and structural variables**	**5**	**9**
**Incorporated analytically**	**2**	**4**
**Collected but not analyzed**	**3**	**6**

Studies (*n* = 54) were assessed for how race/ethnicity was reported and whether analyses considered the impact of additional determinants of bone differences, including puberty, body composition, other biological variables, and social/structural factors. Subcategories list the specific approaches used within each domain. Number of studies (*n*) and percent of total study population (%) are shown.

### The influence of puberty on measurements of BMD

Pubertal development and sex steroid exposure play a central role in bone mineral accrual yet approaches to capturing pubertal progression varied across the 54 included studies. Only 39 studies (72%) accounted for pubertal status in any form (Table 3), most often through Tanner staging, menarcheal status, or bone age. Explicit statistical adjustment for puberty as a covariate was uncommon. Tanner staging was the predominant method, employed in all 39 studies that considered puberty. While Tanner staging provides a standardized assessment of pubertal development based on external physical characteristics—testicular and penile development in those assigned male at birth, and breast development in those assigned female at birth—it remains a semi-subjective measure with limitations. Accurate assessment requires a trained examiner, yet even among trained clinicians, inter-rater variability is well documented.^63^ Self-report or parent-report, although occasionally used, is often unreliable due to the subtle distinctions between stages and the social discomfort of evaluating genital or breast development. Of the 39 studies, 22 (56%) employed direct physical examinations by trained study personnel, while 6 studies restricted their cohorts to a single pubertal stage. For example, 2 studies examined prepubertal Black and White children,^12^^,^^29^ while others focused on adolescents who had already entered or completed puberty.^41^^,^^51^ Though such restriction reduces heterogeneity, it precludes the assessment of bone mineral accrual across the pubertal transition.

Notably, none of the included studies incorporated time since pubertal onset as a covariate. While cumulative sex steroid exposure is an important determinant of bone mineral accrual, accurate assessment of pubertal onset remains challenging. As a result, pediatric DXA studies have largely relied on cross-sectional indicators or surrogate markers, such as Tanner stage, menarcheal status, or bone age.^30^

### Accounting for body composition in measurements of BMD

Body composition strongly influences pediatric bone health. Heavier children typically have higher bone density, with lean mass contributing positively and fat mass—particularly abdominal fat—exerting negative effects.^10^^,^^20^^,^^21^^,^^28^^,^^50^ Despite these well-established associations, many studies of racial differences in bone outcomes did not adjust for body composition.^22–24^^,^^30^^,^^33^ In our review, just over half of the studies considered these factors, and methods of adjustment varied widely. Weight was the most frequently included covariate. Across multiple studies, adjustment for weight reduced or eliminated reported racial differences in bone outcomes, or improved model accuracy.^12^^,^^22^^,^^27^^,^^34^^,^^37^ Several studies also examined lean mass, showing that racial differences in BMC often diminished once differences in muscle mass were incorporated.^20^^,^^30^^,^^32^^,^^33^^,^^51^^,^^60^ Fat mass was less consistently included, but when examined, it similarly weakened observed racial differences.^20–22^^,^^30^

Height was another critical covariate. The introduction of height-adjusted Z-scores provided an important advance, reducing bias when assessing children at the extremes of stature.^44^ Studies incorporating adjustments, whether as covariates or through reference standards, consistently found that accounting for stature reduced—but did not always eliminate—apparent racial differences in bone density.^16^^,^^22^^,^^23^^,^^33^^,^^51^

These findings suggest that much of the variability attributed to race in pediatric bone studies could instead be explained by underlying differences in body size and composition. The inconsistent application of these adjustments across the literature represents a major limitation and raises concern that unmeasured body composition factors may have been misattributed due to intrinsic racial differences.

### Influence of social determinants of health

Social determinants of health exert a profound and multifaceted influence on pediatric bone health outcomes.^64^ Effects vary by skeletal site and are mediated through nutrition, growth, adiposity, pubertal timing, and physical activity. In the U.S., children from lower-income families generally have lower BMD, reflecting reduced stature and lean mass, delayed puberty, and lower calcium intake and activity levels. Large cohort studies illustrate this complexity: one study reported that higher maternal SES was associated with greater stature and bone mass, while lower SES was associated with greater adiposity and bone area, with associations eliminated after adjustment for body size.^64^ Another found that food insecurity was linked to lower calcium intake and reduced BMC in boys, but not girls.^65^ Current household resources were shown to be more predictive of whole-body bone outcomes than early-life SES,^66^ and disparities were most pronounced during puberty.^67^ Together, these findings underscore social determinants of health as critical determinants of bone accrual across childhood and adolescence.

Despite this evidence, structural and social determinants of health remain underexamined in studies of racial and ethnic differences in pediatric bone outcomes. In our review of 54 studies, only a handful acknowledged social factors influencing bone health. Just 2 (~4%) incorporated these variables analytically: one adjusted for SES when examining BMC across racial/ethnic groups and genetic admixture,^21^ and another included parental education in multivariable models of BMD.^16^ Several others collected SES information but did not use it in analyses: 2 employed SES only as a matching criterion,^14^^,^^15^ and 1 recorded parental education but excluded it from outcome models.^34^ Notably, none of the studies considered healthcare access—an essential indicator of socioeconomic position with direct implications for pediatric health. The omission of factors, such as income, education, and healthcare access, highlights a critical gap in the DXA literature: without accounting for these factors, interpretations of bone health disparities across populations risk being incomplete or misleading.

### Physical activity in relation to racial differences in BMD

Out of the 54 studies included in this scoping review, only 8 (15%) explicitly reported data on physical activity (Table 3).^17^^,^^18^^,^^21^^,^^27^^,^^28^^,^^33^^,^^37^^,^^68^ Of these 8 studies, 4 adjusted for physical activity in their analyses of racial differences in BMD or BMC.^21^^,^^28^^,^^33^^,^^68^ The remaining 4 studies collected physical activity data but did not include it as a covariate in their analyses.^17^^,^^18^^,^^27^^,^^37^ Only a small subset of studies accounted for other biological covariates: 6 out of 54 adjusted for nutrition and just 1 adjusted for birth control use.

### Repeated analyses of BMDCS and NHANES

Twelve (23%) of the 54 studies included in this review analyzed data from the same longitudinal Bone Mineral Density in Childhood Study (BMDCS) DXA dataset (Table 1).^22–24^^,^^33^^,^^42–44^^,^^52–55^ Each of these studies pursued distinct analytic aims, such as developing reference curves, evaluating site-specific measures, or testing methodological approaches, yet all relied on the same underlying cohort of ~2000 children. As a result, BMDCS participants are disproportionately represented in the pediatric bone literature. Similarly, 8 (15%) of the 54 studies relied on data from the National Health and Nutrition Examination Survey (NHANES).^32^^,^^46^^,^^47^^,^^50^^,^^56^^,^^57^^,^^59^^,^^60^ While NHANES provides valuable nationally representative data, it includes relatively limited covariates for statistical modeling, which might have attenuated observed racial differences. Taken together, nearly 40% of the studies reviewed draw on only 2 datasets (BMDCS and NHANES). This reliance underscores how heavily the field depends on a narrow set of cohorts, which may constrain the interpretability and generalizability of reported racial differences in pediatric bone outcomes.

## Discussion

Although many studies have reported racial and ethnic differences in BMD, our review highlights substantial methodological variability in how race is defined, and covariates are addressed across analyses. These inconsistencies call into question the reliability and clinical utility of long-standing assumptions about pediatric bone density differences. Because race is recognized as a social construct rather than a biological determinant, reliance on race-stratified DXA reference curves risks interpreting observed group-level differences in bone density as intrinsic skeletal differences, even though studies show that body composition, pubertal timing, and related factors strongly influence these measures and vary across socially defined racial groups.^16^^,^^28^ The impact of race-based pediatric DXA reference curves on differential clinical decision-making or disparities is a topic that merits further evaluation. This concern is reinforced by our findings that reported racial differences in pediatric BMD frequently attenuated or disappeared after adjustment for stature, lean mass, pubertal status, and related covariates, suggesting that race may capture heterogenous underlying influences rather than serving as a mechanistic determinant of bone density. Body composition itself varies across socially defined racial groups, including in BMDCS and NHANES.^16^^,^^60^ These differences likely contribute to observed group-level differences in bone density and are consistent with our findings that adjustment for lean mass, fat mass, or related measures often attenuates reported racial differences. At the same time, multiple studies demonstrate that inclusion of body composition does not uniformly eliminate differences in bone density, suggesting the influence of additional biological, developmental, or social factors.^14^^,^^26^ Together, these findings underscore the analytic complexity of interpreting bone density across racially diverse pediatric populations. One study that identified genetic admixture and body composition as stronger predictors of bone outcomes, than self-reported race, further highlighting the limitations of simplistic racial categorizations.^21^ Even when statistically significant, reported differences between racial groups were modest in magnitude and constrained by limited covariate adjustment, reducing their clinical interpretive relevance and highlighting the need for more objective analytic approaches.

Interpretation of pediatric DXA Z-scores requires careful consideration, as Z-score thresholds alone do not diagnose osteoporosis in children, unlike in adults. Rather, bone density results must be interpreted within a broader clinical context including fracture history, underlying diagnoses, medication exposures, growth patterns, and pubertal status. As a result, patients whose DXA Z-scores fall around the threshold of −2.0 should be interpreted with caution, regardless of race or reference range used. Small shifts in Z-score around this boundary may result in changes in report categorization from “normal” to “abnormal,” or vice versa. Such shifts can occur due to changes in reference datasets or challenges in racial categorization, particularly affecting children of mixed race. These shifts are inherently bidirectional, highlighting the limitation of relying on categorical cutoffs in pediatric DXA interpretation rather than indicating a unique risk to any single reference framework. Both the currently used race-based pediatric DXA reference framework and any future race-neutral reference approaches carry potential clinical limitations. Race-based references rely on biologically imprecise groupings, while race-neutral frameworks may introduce shifts in Z-score classification with downstream clinical implications. These considerations underscore the need for future research in this area to enhance clinical practices and to determine how race-based or race-neutral reference frameworks impact clinical interpretations of pediatric DXA.

In addition, our prior work has identified that practical challenges also arise when race-based pediatric DXA reference curves are applied in clinical settings (Ramadan et al., 2024). Race and ethnicity are typically assigned by self- or parent-report, yet guidance for how these categories should be operationalized in DXA interpretation is limited. As a result, classification may be inconsistent across institutions or over time, particularly for children who do not fit neatly into existing categories. Evidence from broader healthcare settings indicates that collecting race or ethnicity information can be experienced as uncomfortable by some patients and may raise concerns about potential misuse, especially among those with prior experiences of discrimination.^7^^,^^8^ These considerations highlight the importance of clear rationale, consistent implementation, and ongoing evaluation when race is incorporated into clinical algorithms.

If the goal of normalization is to provide patients with a more individualized definition of “normal,” emphasis should instead fall on determinants with direct influence on bone accrual, such as pubertal status, lean mass, and body size. Reliance on these objective measures offers a more precise alternative to poorly defined social categories and aligns with broader efforts in medicine to dismantle race-based algorithms in favor of individualized, biologically, and socially informed care.

This review has limitations. As a scoping study, we did not conduct a formal meta-analysis and cannot assess aggregate effect sizes. By restricting U.S. studies, we aimed to ensure sociocultural comparability, but this decision may have excluded relevant international perspectives and underrepresented null findings. Some included studies examined children with larger body size, sometimes categorized as obesity. Whether increased body size should be considered a disease state or variant of normal growth remains debated but given its rising prevalence and direct relevance to pediatric bone densitometry, we retained such studies. Finally, many analyses drew on large, shared datasets such as NHANES and the BMDCS. Repeated use of the same cohorts raises concerns about sampling bias and constrains generalizability, though these data remain foundational to current pediatric reference standards.

### Conclusion

This scoping review demonstrates that race-based interpretation of pediatric DXA relies on evidence that is inconsistent in its definitions of race and limited in adjusting for biological and social determinants. These limitations underscore the need to reconsider how race and ethnicity are conceptualized in pediatric bone research and clinical practice. In research contexts, studies focused on social determinants of bone density should adopt standardized definitions of race and ethnicity, while genetic studies must avoid using race as a proxy for ancestry and instead rely on direct measures of genetic ancestry when relevant (eg, GWAS and/or direct genetic testing). Clinically, our findings highlight important limitations of race-based pediatric DXA reference curves; however, direct evidence of downstream clinical outcomes or management changes from adopting race-neutral reference data remains limited. In practice, implementation can be inconsistent, and categorization may be especially challenging for children with mixed racial and ethnic identities, raising the possibility of misclassification in routine interpretation. Additional research evaluating the clinical performance of race-neutral reference standards, including their impact on interpretation and management decisions, is needed before such approaches can broadly be adopted in practice.

From a policy perspective, these findings align with broader efforts to reevaluate race-based algorithms in medicine and underscore the need for development, validation, and dissemination of race-neutral reference standards. Recent work introducing race-neutral pediatric DXA reference data is a significant advance in this direction. Zemel and colleagues demonstrated that fracture risk can be predicted without recourse to racial categories.^16^^,^^33^

Transitioning toward race-neutral, equity-informed standards represents an essential step toward improving the accuracy and fairness of pediatric DXA interpretation. Future work should prioritize validation in diverse cohorts and integration into ISCD guidelines. These measures will be critical to advancing bone health assessment toward greater precision and equity.

## Supplementary Material

Supplementary_Table_1_ziag028

Supplementary_Table_2_ziag028

## Data Availability

No new data were generated or analyzed in support of this research. All data included in this study are derived from previously published articles, which are cited within the manuscript.
