# Supplementary material for: Rethinking race-based interpretation in pediatric densitometry: a scoping review
Source: JBMR Plus. 2026 Mar 12;10(4):ziag028. doi: 10.1093/jbmrpl/ziag028 (PMC13007872; doi:10.1093/jbmrpl/ziag028)
Supplement: Supplementary_Table_1_ziag028 [file supplementary_table_1_ziag028.docx]

| PubMed | (("Absorptiometry, Photon"[mesh] OR "Photon Absorptiometry"[tiab] OR "X Ray Absorptiometry"[tiab] OR "X Ray Absorptiometry"[tiab] OR "Xray Absorptiometry"[tiab] OR "Radiographic Absorptiometry"[tiab] OR "X Ray Densitometry"[tiab] OR "X Ray Photodensitometry"[tiab] OR "DXA Scan*"[tiab] OR "DEXA Scan*"[tiab] OR densitometry[tiab]) AND ("Bone Density"[mesh] OR "Bone Densit*"[tiab] OR "Bone Mineral Densit*"[tiab] OR "Bone Mineral Content*"[tiab] OR BMD[tiab] OR "bone mass"[tiab] OR "osseous densit*"[tiab] OR osteoporosis[mesh] OR osteoporosis[tiab] OR osteoporotic[tiab] OR "pathologic decalcification"[tiab] OR frax[tiab] OR "fracture risk"[tiab] OR "risk of fracture"[tiab] OR "bone fragility"[tiab] OR "fracture prone"[tiab:~0] OR "fracture proneness"[tiab:~0] OR "prone to fracture"[tiab:~0] OR "fracture propensity"[tiab] OR "Bone Diseases, Metabolic"[mesh] OR osteopenia[tiab] OR "metabolic bone disease*"[tiab])) AND (("Racial Groups"[mesh] OR "Population Groups"[mesh] OR Ethnicity[mesh] OR White[mesh] OR "White People"[mesh] OR "Native Hawaiian or Other Pacific Islander"[mesh] OR "Oceanians"[mesh] OR "Black or African American"[mesh] OR "African People"[mesh] OR Asian[mesh] OR "Asian People"[mesh] OR "American Indian or Alaska Native"[mesh] OR "Hispanic or Latino"[mesh] OR "Race Factors"[mesh] OR "middle eastern people"[mesh] OR "Middle Eastern and North Africans"[mesh] OR "arabs"[mesh] OR "caribbean people"[mesh] OR "central american people"[mesh] OR "european people"[mesh] OR "south american people"[mesh] OR "north american people"[mesh] OR "african people"[mesh] OR "racial group*"[tiab] OR "race group*"[tiab] OR race[tiab] OR racial[tiab] OR "non black"[tiab] OR "Population Group*"[tiab] OR "ancestry group*"[tiab] OR ethnic*[tiab] OR white[tiab] OR whites[tiab] OR caucasian*[tiab] OR "european ancestry"[tiab] OR "european descent"[tiab] OR Hawaiian*[tiab] OR "pacific island*"[tiab] OR oceanean*[tiab] OR aboriginal[tiab] OR Australasian*[tiab] OR black[tiab] OR blacks[tiab] OR "african american*"[tiab] OR "afro american*"[tiab] OR "african ancestry"[tiab] OR "african descent"[tiab] OR asian[tiab] OR asians[tiab] OR "native american*"[tiab] OR "american native*"[tiab] OR "american indian*"[tiab] OR indiginous[tiab] OR hispanic[tiab] OR latin*[tiab] OR "race factor*"[tiab] OR "racial factor*"[tiab] OR "middle eastern"[tiab] OR "north african*"[tiab] OR "MENA people"[tiab:~0] OR arab[tiab] OR arabs[tiab] OR "caribbean people"[tiab] OR "caribbean person"[tiab:~0] OR "caribbean persons"[tiab:~0] OR caribbeans[tiab] OR "west indian*"[tiab] OR "central american*"[tiab] OR european*[tiab] OR "south american*"[tiab] OR "north american*"[tiab] OR african*[tiab] OR "mixed ancestry"[tiab] OR biracial[tiab] OR "multi-ancestry"[tiab] OR "multi ancestral"[tiab] OR "mixed race"[tiab] OR multiracial[tiab] OR "admixed ancestry"[tiab] OR "mixed racial"[tiab] OR "unknown race"[tiab]) OR ("Reference Values"[mesh] OR "reference value*"[tiab] OR "reference interval*"[tiab] OR "reference range*"[tiab] OR "reference database*"[tiab] OR normaliz*[tiab] OR normative[tiab] OR "z score*"[tiab] OR NHANES[tiab] OR "national health and nutrition examination survey"[tiab]))) AND (Pediatrics[mesh] OR "Infant, newborn"[mesh] OR Infant[mesh] OR "Child, preschool"[mesh] OR Child[mesh] OR Adolescent[mesh] OR pediatric*[tiab] OR newborn*[tiab] OR neonat*[tiab] OR infant*[tiab] OR toddler*[tiab] OR baby[tiab] OR babies[tiab] OR school age*[tiab] OR preschool age*[tiab] OR "pre school age"[tiab] OR child*[tiab] OR adolescen*[tiab] OR teen*[tiab] OR youth*[tiab]) AND english[language] |
| --- | --- |
| EMBASE | (("dual energy X ray absorptiometry"/exp OR "Photon Absorptiometry":ab,ti OR "X Ray Absorptiometry":ab,ti OR "X Ray Absorptiometry":ab,ti OR "Xray Absorptiometry":ab,ti OR "Radiographic Absorptiometry":ab,ti OR "X Ray Densitometry":ab,ti OR "X Ray Photodensitometry":ab,ti OR "DXA Scan*":ab,ti OR "DEXA Scan*":ab,ti OR densitometry:ab,ti) AND ("Bone Density"/exp OR "Bone Densit*":ab,ti OR "Bone Mineral Densit*":ab,ti OR "Bone Mineral Content*":ab,ti OR BMD:ab,ti OR "bone mass":ab,ti OR "osseous densit*":ab,ti OR osteoporosis/exp OR osteoporosis:ab,ti OR osteoporotic:ab,ti OR "pathologic decalcification":ab,ti OR “frax tool”/exp OR frax:ab,ti OR "fracture risk":ab,ti OR "risk of fracture":ab,ti OR “bone fragility”/exp OR "bone fragility":ab,ti OR "fracture prone":ab,ti OR "fracture proneness":ab,ti OR "prone to fracture":ab,ti OR "fracture propensity":ab,ti OR "osteopenia"/exp OR osteopenia:ab,ti OR "metabolic bone disease*":ab,ti) AND (("ancestry group"/exp OR "citizen group"/exp OR Ethnicity/exp OR caucasian/exp OR "Oceanic ancestry group"/exp OR "black person"/exp OR "African People"/exp OR Asian/exp OR "American Indian"/exp OR "Hispanic"/exp OR "Race"/exp OR "middle eastern person"/exp OR "Middle Eastern/North African"/exp OR "arab"/exp OR "caribbean person"/exp OR "central american "/exp OR "european"/exp OR "south american"/exp OR "north american"/exp OR "african"/exp OR “people of mixed ancestry”/exp OR "racial group*":ab,ti OR "race group*":ab,ti OR race:ab,ti OR racial:ab,ti OR "non black":ab,ti OR "Population Group*":ab,ti OR "ancestry group*":ab,ti OR ethnic*:ab,ti OR white:ab,ti OR whites:ab,ti OR caucasian*:ab,ti OR "european ancestry":ab,ti OR "european descent":ab,ti OR Hawaiian*:ab,ti OR "pacific island*":ab,ti OR oceanean*:ab,ti OR aboriginal:ab,ti OR Australasian*:ab,ti OR black:ab,ti OR blacks:ab,ti OR "african american*":ab,ti OR "afro american*":ab,ti OR "african ancestry":ab,ti OR "african descent":ab,ti OR asian:ab,ti OR asians:ab,ti OR "native american*":ab,ti OR "american native*":ab,ti OR "american indian*":ab,ti OR indiginous:ab,ti OR hispanic:ab,ti OR latin*:ab,ti OR "race factor*":ab,ti OR "racial factor*":ab,ti OR "middle eastern":ab,ti OR "north african*":ab,ti OR "MENA people":ab,ti OR arab:ab,ti OR arabs:ab,ti OR "caribbean people":ab,ti OR "caribbean person":ab,ti OR "caribbean persons":ab,ti OR caribbeans:ab,ti OR "west indian*":ab,ti OR "central american*":ab,ti OR european*:ab,ti OR "south american*":ab,ti OR "north american*":ab,ti OR african*:ab,ti OR "mixed ancestry":ab,ti OR biracial:ab,ti OR "multi-ancestry":ab,ti OR "multi ancestral":ab,ti OR "mixed race":ab,ti OR multiracial:ab,ti OR "admixed ancestry":ab,ti OR "mixed racial":ab,ti OR "unknown race":ab,ti) OR ("Reference Value"/exp OR “z score”/exp OR "reference value*":ab,ti OR "reference interval*":ab,ti OR "reference range*":ab,ti OR "reference database*":ab,ti OR normaliz*:ab,ti OR normative:ab,ti OR "z score*":ab,ti OR NHANES:ab,ti OR "national health and nutrition examination survey":ab,ti)) AND (Pediatrics/exp OR "newborn"/exp OR Infant/exp OR "toddler"/exp OR Child/exp OR Adolescence/exp OR pediatric*:ab,ti OR newborn*:ab,ti OR neonat*:ab,ti OR infant*:ab,ti OR toddler*:ab,ti OR baby:ab,ti OR babies:ab,ti OR school age*:ab,ti OR preschool age*:ab,ti OR "pre school age":ab,ti OR child*:ab,ti OR adolescen*:ab,ti OR teen*:ab,ti OR youth*:ab,ti) AND English:la) NOT “conference abstract”:it |
| Web of Science | ((TI=("Photon Absorptiometry" OR "X Ray Absorptiometry" OR "X Ray Absorptiometry" OR "Xray Absorptiometry" OR "Radiographic Absorptiometry" OR "X Ray Densitometry" OR "X Ray Photodensitometry" OR "DXA Scan*" OR "DEXA Scan*" OR densitometry) OR AB=("Photon Absorptiometry" OR "X Ray Absorptiometry" OR "X Ray Absorptiometry" OR "Xray Absorptiometry" OR "Radiographic Absorptiometry" OR "X Ray Densitometry" OR "X Ray Photodensitometry" OR "DXA Scan*" OR "DEXA Scan*" OR densitometry)) AND (TI=("Bone Densit*" OR "Bone Mineral Densit*" OR "Bone Mineral Content*" OR BMD OR "bone mass" OR "osseous densit*" OR osteoporosis OR osteoporotic OR "pathologic decalcification" OR frax OR "fracture risk" OR "risk of fracture" OR "bone fragility" OR "fracture prone" OR "fracture proneness" OR "prone to fracture" OR "fracture propensity" OR osteopenia OR "metabolic bone disease*") OR AB=("Bone Densit*" OR "Bone Mineral Densit*" OR "Bone Mineral Content*" OR BMD OR "bone mass" OR "osseous densit*" OR osteoporosis OR osteoporotic OR "pathologic decalcification" OR frax OR "fracture risk" OR "risk of fracture" OR "bone fragility" OR "fracture prone" OR "fracture proneness" OR "prone to fracture" OR "fracture propensity" OR osteopenia OR "metabolic bone disease*")) AND (TI=("racial group*" OR "race group*" OR race OR racial OR "non black" OR "Population Group*" OR "ancestry group*" OR ethnic* OR white OR whites OR caucasian* OR "european ancestry" OR "european descent" OR Hawaiian* OR "pacific island*" OR oceanean* OR aboriginal OR Australasian* OR black OR blacks OR "african american*" OR "afro american*" OR "african ancestry" OR "african descent" OR asian OR asians OR "native american*" OR "american native*" OR "american indian*" OR indiginous OR hispanic OR latin* OR "race factor*" OR "racial factor*" OR "middle eastern" OR "north african*" OR "MENA people" OR arab OR arabs OR "caribbean people" OR "caribbean person" OR "caribbean persons" OR caribbeans OR "west indian*" OR "central american*" OR european* OR "south american*" OR "north american*" OR african* OR "mixed ancestry" OR biracial OR "multi-ancestry" OR "multi ancestral" OR "mixed race" OR multiracial OR "admixed ancestry" OR "mixed racial" OR "unknown race") OR AB=("racial group*" OR "race group*" OR race OR racial OR "non black" OR "Population Group*" OR "ancestry group*" OR ethnic* OR white OR whites OR caucasian* OR "european ancestry" OR "european descent" OR Hawaiian* OR "pacific island*" OR oceanean* OR aboriginal OR Australasian* OR black OR blacks OR "african american*" OR "afro american*" OR "african ancestry" OR "african descent" OR asian OR asians OR "native american*" OR "american native*" OR "american indian*" OR indiginous OR hispanic OR latin* OR "race factor*" OR "racial factor*" OR "middle eastern" OR "north african*" OR "MENA people" OR arab OR arabs OR "caribbean people" OR "caribbean person" OR "caribbean persons" OR caribbeans OR "west indian*" OR "central american*" OR european* OR "south american*" OR "north american*" OR african* OR "mixed ancestry" OR biracial OR "multi-ancestry" OR "multi ancestral" OR "mixed race" OR multiracial OR "admixed ancestry" OR "mixed racial" OR "unknown race")) AND (TI=(pediatric* OR newborn* OR neonat* OR infant* OR toddler* OR baby OR babies OR school age* OR preschool age* OR "pre school age" OR child* OR adolescen* OR teen* OR youth*) OR AB=(pediatric* OR newborn* OR neonat* OR infant* OR toddler* OR baby OR babies OR school age* OR preschool age* OR "pre school age" OR child* OR adolescen* OR teen* OR youth*)) AND LA=(English)) NOT DT=(Meeting Abstract) |
| CINAHL | (MH "absorbtiometry, photon” OR TI ("Photon Absorptiometry" OR "X Ray Absorptiometry" OR "X Ray Absorptiometry" OR "Xray Absorptiometry" OR "Radiographic Absorptiometry" OR "X Ray Densitometry" OR "X Ray Photodensitometry" OR "DXA Scan*" OR "DEXA Scan*" OR densitometry) OR AB ("Photon Absorptiometry" OR "X Ray Absorptiometry" OR "X Ray Absorptiometry" OR "Xray Absorptiometry" OR "Radiographic Absorptiometry" OR "X Ray Densitometry" OR "X Ray Photodensitometry" OR "DXA Scan*" OR "DEXA Scan*" OR densitometry)) AND (MH ("Bone Density" OR osteoporosis OR “bone diseases, metabolic”) OR TI ("Bone Densit*" OR "Bone Mineral Densit*" OR "Bone Mineral Content*" OR BMD OR "bone mass" OR "osseous densit*" OR osteoporosis OR osteoporotic OR "pathologic decalcification" OR frax OR "fracture risk" OR "risk of fracture" OR "bone fragility" OR "fracture prone" OR "fracture proneness" OR "prone to fracture" OR "fracture propensity" OR osteopenia OR "metabolic bone disease*") OR AB ("Bone Densit*" OR "Bone Mineral Densit*" OR "Bone Mineral Content*" OR BMD OR "bone mass" OR "osseous densit*" OR osteoporosis OR osteoporotic OR "pathologic decalcification" OR frax OR "fracture risk" OR "risk of fracture" OR "bone fragility" OR "fracture prone" OR "fracture proneness" OR "prone to fracture" OR "fracture propensity" OR osteopenia OR "metabolic bone disease*")) AND (MH ("ethnic groups” OR “white persons” OR Australasians OR "black persons" OR Asians OR "indigenous peoples" OR "Hispanic Americans" OR "Race factors" OR "middle eastern persons" OR arabs OR "caribbean persons" OR "central americans" OR "europeans" OR "south americans" OR "north americans" OR africans OR “multiracial persons”) OR TI ("racial group*" OR "race group*" OR race OR racial OR "non black" OR "Population Group*" OR "ancestry group*" OR ethnic* OR white OR whites OR caucasian* OR "european ancestry" OR "european descent" OR Hawaiian* OR "pacific island*" OR oceanean* OR aboriginal OR Australasian* OR black OR blacks OR "african american*" OR "afro american*" OR "african ancestry" OR "african descent" OR asian OR asians OR "native american*" OR "american native*" OR "american indian*" OR indiginous OR hispanic OR latin* OR "race factor*" OR "racial factor*" OR "middle eastern" OR "north african*" OR "MENA people" OR arab OR arabs OR "caribbean people" OR "caribbean person" OR "caribbean persons" OR caribbeans OR "west indian*" OR "central american*" OR european* OR "south american*" OR "north american*" OR african* OR "mixed ancestry" OR biracial OR "multi-ancestry" OR "multi ancestral" OR "mixed race" OR multiracial OR "admixed ancestry" OR "mixed racial" OR "unknown race") OR AB ("racial group*" OR "race group*" OR race OR racial OR "non black" OR "Population Group*" OR "ancestry group*" OR ethnic* OR white OR whites OR caucasian* OR "european ancestry" OR "european descent" OR Hawaiian* OR "pacific island*" OR oceanean* OR aboriginal OR Australasian* OR black OR blacks OR "african american*" OR "afro american*" OR "african ancestry" OR "african descent" OR asian OR asians OR "native american*" OR "american native*" OR "american indian*" OR indiginous OR hispanic OR latin* OR "race factor*" OR "racial factor*" OR "middle eastern" OR "north african*" OR "MENA people" OR arab OR arabs OR "caribbean people" OR "caribbean person" OR "caribbean persons" OR caribbeans OR "west indian*" OR "central american*" OR european* OR "south american*" OR "north american*" OR african* OR "mixed ancestry" OR biracial OR "multi-ancestry" OR "multi ancestral" OR "mixed race" OR multiracial OR "admixed ancestry" OR "mixed racial" OR "unknown race") OR MH "Reference Values" OR TI ("reference value*" OR "reference interval*" OR "reference range*" OR "reference database*" OR normaliz* OR normative OR "z score*" OR NHANES OR "national health and nutrition examination survey") OR AB ("reference value*" OR "reference interval*" OR "reference range*" OR "reference database*" OR normaliz* OR normative OR "z score*" OR NHANES OR "national health and nutrition examination survey")) AND (MH (Pediatrics OR "infant, newborn" OR Infant OR "child, preschool" OR Child OR Adolescence) OR TI (pediatric* OR newborn* OR neonat* OR infant* OR toddler* OR baby OR babies OR school age* OR preschool age* OR "pre school age" OR child* OR adolescen* OR teen* OR youth*) OR AB (pediatric* OR newborn* OR neonat* OR infant* OR toddler* OR baby OR babies OR school age* OR preschool age* OR "pre school age" OR child* OR adolescen* OR teen* OR youth*)) AND LA English |
