# Supplementary material for: Rethinking race-based interpretation in pediatric densitometry: a scoping review
Source: JBMR Plus. 2026 Mar 12;10(4):ziag028. doi: 10.1093/jbmrpl/ziag028 (PMC13007872; doi:10.1093/jbmrpl/ziag028)
Supplement: Supplementary_Table_2_ziag028 [file supplementary_table_2_ziag028.docx]

| **No differences (n=12)** |
| --- |
| 1. **Morrison et al. (1996)** ^†^ studied 98 girls (ages 4–10) and found no significant racial differences in bone mineral density; Black girls had only slightly higher bone mass, which was not statistically significant. Analyses focused primarily on resting energy expenditure rather than bone outcomes. 2. **Barbeau et al. (1999)** ^†^ studied 71 children (ages 7–11) and found that bone mass response to physical training did not differ significantly between Black and White participants. In regression models controlling for sex, age, baseline body composition, diet, and activity, ethnicity was not a significant predictor of changes in BMC or aBMD. 3. **Tershakovec et al. (2002)** ^*^ studied 203 children (ages 7–17) with high BMI (70 Black, 133 White) and found no significant racial differences in whole-body bone mass. Bone outcomes were measured by DXA but analyzed only as part of body composition in a study focused on resting energy expenditure. 4. **Afghani & Goran (2006)** ^a^ studied 181 children (ages 8–13) and found no significant overall differences in bone mass between races. However, bone mass was more negatively influenced by subcutaneous fat in White children and by visceral fat in Black children. 5. **Dorsey et al. (2010)**^a^ studied 175 children (ages 6–18) and found that Hispanic ethnicity was independently associated with greater whole-body BMC after adjustment for sex, age, height, adiposity, and skeletal muscle mass. No significant differences in BMC were observed for Black, White, or Asian participants. 6. **Casazza et al. (2010)** ^a^ studied 270 peripubertal children (ages 7–12; 129 White, 89 Black, 52 Hispanic) and found that apparent racial/ethnic differences in bone mineral content were fully explained by genetic admixture and adiposity. After adjusting for admixture proportions, Tanner stage, and body composition, self-reported race/ethnicity was no longer a significant predictor of bone outcomes. 7. **Short et al. (2011)** ^†a^ studied 1,889 children and adolescents (ages 5–20) in the BMDCS and found that Black participants had higher lumbar spine aBMD than non-Black peers, but this difference was largely attenuated after adjustment for weight, height, percent body fat, and pubertal maturity. Only the lumbar spine was analyzed, and lifestyle or social factors were not included. 8. **Short et al. (2015)** ^†a^ studied 2,014 participants in the BMDCS and found that Black children had higher unadjusted BMC and aBMD than non-Black peers, but these differences were attenuated and explained almost entirely by age, sex, height, weight, and body fat. The authors concluded that anthropometric models provided a more accurate basis for bone Z-scores than race-based models. 9. **Lappe et al. (2015)** ^a^ studied 1,743 children in the BMDCS and focused on BMC accrual rather than absolute aBMD. Black youth accrued slightly more BMC than non-Black peers at some pubertal stages, but physical activity (both groups) and calcium intake (non-Black girls only) were the key predictors. Race was not retained as an independent predictor, and no claims were made regarding racial differences in aBMD. 10. **Cattran et al. (2015)** ‡ studied 227 girls (ages 6–13) and found no significant racial differences in lumbar spine BMD Z-scores between Black and White participants. Instead, earlier puberty timing was the main factor associated with higher BMD. Regression models adjusting for pubertal stage, race, and family history of breast cancer confirmed that race was not a significant predictor. 11. **Broadney et al. (2018)** ^a^ studied 594 children (ages 5–18) in an analysis primarily focused on resting energy expenditure. Bone mass was measured as part of DXA body composition; Black participants had higher bone mass than White peers, but bone was not analyzed as an outcome and racial differences in bone density were not interpreted. 12. **Campoverde Reyes et al. (2020)** ^‡^ studied 46 adolescents with high BMI (30 White, 16 Black) and found no significant racial differences in aBMD measured by DXA. 13. **Kalkwarf et al. (2022;**TBS study**)** ^‡^ analyzed 2,014 children in the BMDCS and found no differences in TBS between those categorized as Black versus non-Black peers. . |
| **Mixed findings and/or attenuated differences after covariate analysis (n=10)** |
| 1. **Moro et al. (1996)** ^†^ studied 375 adolescents and found ethnic differences in femoral bone measures at certain pubertal stages (e.g., Black boys lower than Asian/Hispanic boys, Black girls higher than others, Asian girls lower than Black/White girls), but body mass explained 69–87% of the variance, and once body mass was included, ethnicity was no longer an independent predictor. 2. **Yanovski et al. (1996)** ^†^ studied 40 prepubertal girls (ages 7–10) matched for age, BMI, Tanner stage, bone age, and SES, and found that Black girls had higher lumbar spine, pelvis, and leg aBMD than White peers, while total-body aBMD showed no racial difference. Differences persisted at some sites after matching, but lean and fat mass, diet, activity, and social factors were not analyzed. 3. **Nelson et al. (1997)** ^a^ studied 773 third- and fourth-grade children (ages 8–10) and found that Black children had higher whole-body BMC and aBMD than White peers, with greater annual gains in bone and lean mass; regression models showed that weight explained most of the variance, and racial differences were largely attenuated but not fully eliminated, while pubertal stage and social factors were not analyzed. 4. **Yanovski et al. (2000)** ^†a^ studied 118 pre/early pubertal girls (59 Black, 59 White) matched for age, BMI, Tanner stage, and SES, and found that Black girls had significantly higher total-body BMC and aBMD than White peers; however, once IGF-1 was included in regression models alongside height and weight, race was no longer an independent predictor. Lean and fat mass, diet, and social factors were not analyzed. 5. **Wright et al. (2002)** ^†a^ studied 31 prepubertal boys (ages 9–11) and found that Black boys had higher lumbar spine and femoral neck aBMD than White peers, while total-body aBMD showed no racial difference; differences at spine and hip persisted after adjustment for age, height, weight, and GH secretion, but lean and fat mass, diet, and social factors were not analyzed. 6. **Hui et al. (2003)** ^a^ studied 232 children (ages 4–16; equal numbers of Black and White, boys and girls) and found that Black children had higher whole-body BMC but not spine BMC after adjusting for age, sex, and Tanner stage. The whole-body BMC difference was reduced and became nonsignificant after further adjustment for height, weight, body composition, and biochemical markers of bone turnover, indicating that most observed racial differences were attributable to body build and metabolic factors. 7. **Ackerman et al. (2006)** ^a^ studied 926 healthy children (ages 6–18) and found that Black girls and boys had higher mean BMC than White and Asian peers; in regression models including lean mass, fat mass, height, age, and puberty, Black ethnicity remained a significant positive predictor of BMC but with a smaller effect size, indicating that much of the difference was explained by body size and composition, although not entirely eliminated. Because the study analyzed BMC rather than aBMD or BMAD, the results remain partly size-dependent despite adjustment for height. 8. **Hui et al. (2010)** ^a^ studied 188 Black and White children (ages 5–15; followed up to 4 years) and analyzed velocities of bone mineral accrual rather than absolute bone mass. Black children had higher accrual rates prepubertally and postpubertally, while White children had higher accrual mid-puberty. After adjusting for skeletal size (height, height velocity, bone area), racial differences in accrual rates were no longer significant. Lean mass, diet, and social factors were not included. 9. **Duran et al. (2018)** ^‡^ analyzed NHANES DXA data (1999–2004) and found that non-Hispanic Black males had higher whole-body (TBLH) BMC for lean body mass than White or Mexican American males, whereas females showed little racial difference; the effects of age, height, and body fat on the muscle–bone relationship were otherwise comparable across racial/ethnic groups. 10. **Zemel et al. (2025)** ^‡^ reanalyzed 2,014 children in the BMDCS and found that Black participants appeared to have higher BMC and aBMD than non-Black peers when race-specific reference curves were used. However, these differences were largely attenuated when applying race-neutral standards adjusted for age, sex, height, and body size. Race-neutral curves aligned Black children’s values closer to the population median and performed equally well in predicting fracture risk. |
| **Persistent differences after covariate analysis (n= 26)** |
| 1. **Ellis et al. (1997)** ^a^ studied 313 girls (ages 3–18) and found that Black participants had significantly higher whole-body BMC (not aBMD) and lean tissue mass than White peers after adjusting for age, weight, and height, while Hispanic girls did not differ. The bone–lean mass relationship was consistent across ethnic groups. Only whole-body BMC was measured. 2. **Nelson & Barondess (1997)** ^†a^ studied 734 prepubertal children (mean age ~9) and found that Black and Middle Eastern (Chaldean) participants had significantly higher whole-body BMC and BMC/height than White peers. Differences were partly explained by body size (height, lean, and fat mass) but persisted after adjustment. Social factors were not assessed. 3. **Bachrach et al. (1999)** ^†^ studied 423 healthy youth (ages 9–25) and found that Black participants had consistently higher aBMD and BMAD than non-Black peers at the spine, hip, femoral neck, and whole body. Differences persisted after adjusting for pubertal stage and body size, though lean mass, diet, activity, and SES were not included. 4. **Wang et al. (1999)** ^†a^ studied 304 children (ages 6–18; 141 White, 101 Black, 62 Asian) and found that Black participants had higher total-body BMC than White and Asian peers, with differences most pronounced in pubertal Black girls. Differences persisted after adjustment for age, weight, sex, and pubertal stage, but lean mass, fat mass, diet, and social factors were not included. 5. **Horlick et al. (2000)** ^†^ studied 336 prepubertal children (ages 6–11; Asian, Black, and White) and found that Black participants had significantly higher total-body BMC than non-Black peers after adjusting for age, height, weight, and bone area (difference ≈ 31 g, p = 0.001). Asians and Whites did not differ. Racial differences were partly explained by body size. 6. **Bray et al. (2001)** ^†a^ studied 129 pre/early pubertal children (ages 10–12) and found that Black participants had significantly higher whole-body BMC and aBMD than White peers, independent of sex and body fat group. 7. **Henderson et al. (2002)** ^†^ studied 256 children (ages 3–18.5) and found that Black participants (n = 25) had higher distal femur aBMD than peers across all regions and ages. Models adjusted only for age; pubertal stage, body composition, and social factors were not included. 8. **Wong et al. (2002)** ^a^ studied 141 girls and adolescents (ages 9–17) and found that Black participants had significantly higher BMC and BMC/FFM than White peers. Bone was a secondary outcome in this study of body fat measurement; covariates such as lean and fat mass, diet, activity, and SES were not analyzed. 9. **Horlick et al. (2004)** ^†a^ studied 1,218 children (ages 6–18; Asian, Black, Hispanic, and White) and found that Black participants had higher total-body BMC and aBMD than other groups. Differences remained significant after adjusting for age, height, weight, and bone area. 10. **Cromer et al. (2004)** ^†^ studied 422 adolescent girls (ages 12–18) and found that Black participants had significantly higher lumbar spine and femoral neck aBMD and BMAD than non-Black peers. Differences remained significant after adjusting for age and weight. The study population was limited to later puberty stages; lean/fat mass, diet, activity, and SES were not analyzed. 11. **Kalkwarf et al. (2007)** ^‡^ studied 1,554 children (ages 6–16) in the BMDCS and found that Black participants had higher BMC and aBMD than non-Black peers at the whole body, lumbar spine, hip, femoral neck, and forearm. Differences persisted across ages and sexes. Puberty, body composition, diet, and SES were not included. 12. **Zemel et al. (2009)** ^‡^ studied 821 children (ages 5–18) and developed new reference curves for lateral distal femur aBMD. Black participants had higher aBMD than non-Black peers, leading the authors to recommend separate race-specific curves. Models did not adjust for puberty, body composition, diet, activity, or SES. 13. **Zemel et al. (2011)** ^‡^ studied 2,014 children in the BMDCS and found that Black participants had higher BMC and aBMD than non-Black peers at all skeletal sites. Differences persisted after adjustment for age, sex, and height-for-age, with median Black values corresponding to the 70th–81st percentile on non-Black curves. Lean and fat mass, diet, activity, and SES were not included. 14. **Gutin et al. (2011)** ^a^ studied 660 adolescents (ages 14–18; 362 White, 298 Black) and found that Black participants had significantly higher total body BMC than White peers. These differences persisted after adjustment for diet and physical activity, but other covariates such as pubertal stage, lean and fat mass, and SES were not included. 15. **Looker et al. (2012)** ^†^ analyzed NHANES 2005–2008 DXA data from 15,026 participants (ages 8+) and found that non-Hispanic Black participants had significantly higher lumbar spine (~6%) and femoral neck (~9–10%) aBMD than White peers across nearly all age groups. Mexican American participants showed smaller, mixed differences. Analyses adjusted only for age and sex. 16. **Looker et al. (2013)** ^†^ analyzed NHANES 1999–2006 DXA data from 22,667 participants (ages 8+) and found that non-Hispanic Black participants had significantly higher total-body aBMD than White peers across nearly all age and sex groups, while Mexican American participants had similar or lower values depending on subgroup. Differences were consistent across most subregions. Analyses adjusted only for age and sex. 17. **Newton et al. (2013)** ^a^ studied 59 early-pubertal girls (ages 4–10; 25 Black, 34 White) and found that Black participants had significantly higher total-body BMC. No other skeletal sites were analyzed. Models adjusted for age and height, but not puberty (though most were Tanner I-2). Lean mass, diet, activity, and SES were not included. 18. **Hanks et al. (2015)** ^a^ studied 37 pre/early pubertal boys (ages 7–12; Tanner stage <3) and found that Black participants had significantly higher whole-body BMC than White peers. Differences persisted after adjusting for percent body fat. Lean mass, later pubertal stages, and social factors were not analyzed. 19. **Gállego Suárez et al. (2017)** ^†^ analyzed NHANES DXA data from 8,348 children and adolescents and found that Black participants had significantly higher total-body, pelvic, and lumbar spine aBMD than White and Hispanic peers. Differences of ~5–8% persisted after adjustment for age, sex, and lean mass. 20. **Misra et al. (2017)** ^‡^ studied 60 adolescent girls (ages 14–21; 35 White, 15 Asian American, 10 Black) and found that Black participants had higher lumbar spine, femoral neck, and whole-body aBMD and BMAD compared with White and Asian peers. At the distal radius, Black girls also had greater cortical perimeter, cortical thickness, trabecular thickness, trabecular vBMD, and higher estimated stiffness and failure load; many of these persisted after adjustment, while differences at the distal tibia were nonsignificant. 21. **McCormack et al. (2017)** ^‡^ studied 2,014 BMDCS participants and found that Black children had greater BMC at the whole body, spine, hip, femoral neck, and radius, and reached peak BMC accrual at younger ages than non-Black peers. Differences persisted after adjusting for age, sex, and height, but lean/fat mass, diet, activity, and SES were not included. 22. **Kindler et al. (2019)** ^‡^ studied 2,014 BMDCS participants (ages 5–19) and found that Black children had higher lumbar spine BMAD than non-Black peers across ages. Differences persisted after adjustment for age and height. Only the spine was analyzed; lean/fat mass, diet, activity, and SES were not included. 23. **Kindler et al. (2020)** ^‡^ studied 1,554 BMDCS participants (ages 5–19) and found that Black participants had higher ultradistal radius aBMD than non-Black peers across both sexes. Differences persisted after adjusting for age and forearm length. Lean/fat mass, diet, activity, and SES were not included. 24. **Kalkwarf et al. (2022)** ^‡^ studied 483 children (ages 1–5; linked to BMDCS for ages 5–8.9) and found that Black participants had higher BMC and aBMD than non-Black peers at all skeletal sites. Differences were largest at whole body and distal forearm and smaller at the spine. Models adjusted for age, sex, and height-for-age, but not body composition, diet, or SES. 25. **Schafmeyer et al. (2022)**  ^‡^ spanic Black participants had consistently higher lower-limb BMC and aBMD than White and Mexican American peers across ages and sexes. Models adjusted only for age and sex. 26. **Schafmeyer et al. (2024)** ^‡^ analyzed NHANES 2015–2018 DXA data from children (ages 8–20) and found that non-Hispanic Black participants had consistently higher lower-limb BMC and aBMD than White and Mexican American peers. Differences persisted after adjustment for BMI, but other covariates were not included. 27. **Gordon et al. (2024)** ^‡^ studied 557 adolescents and found that non-Hispanic Black participants had higher whole-body aBMD than White peers. Genetic ancestry (PC1) was a stronger predictor than self-identified race or skin tone. |
| **Statistics not performed (descriptive only) (n=4)** |
| 1. **Litaker et al. (2003)** ^†^ compared DXA measurements from pencil-beam and fan-beam Hologic scanners in 219 adolescents (117 Black, 102 White; ages 13–18) and found that the QDR-1000 consistently gave higher BMC and aBMD values than the QDR-4500; descriptively, Black adolescents had higher BMC and aBMD than White adolescents, but the study did not test these racial differences statistically, focusing instead on scanner-related variability. 2. **Kelly et al. (2009)** ^‡^analyzed NHANES 1999–2004 DXA data (ages 8–20) and generated whole-body and subtotal reference curves for BMC and aBMD stratified by sex and race/ethnicity (White, Black, Mexican American). Black children consistently had higher values than White and Mexican American peers, but the study did not perform statistical tests of significance between groups (no p-values reported). Important covariates such as pubertal stage, diet, activity, and SES were not included. 3. **Fan et al. (2014)** ^‡^ analyzed NHANES 1999–2004 DXA data (n = 8,056 under age 20) and generated whole-body reference curves for BMC and aBMD on GE Lunar systems. Race- and sex-specific percentile tables showed that Black children and adolescents consistently had higher total-body BMC and aBMD than White peers, with Mexican American youth generally intermediate. The analysis was descriptive only; no statistical models or covariates such as pubertal stage, lean and fat mass, diet, activity, or SES were included. 4. **Shypailo & Wong (2020)** ^a^ created fat-mass reference curves for 1,079 children (ages 2–21). Whole-body BMC was reported descriptively in Table 2, with Black children having higher values than White or Hispanic peers, but BMC was not analyzed statistically or modeled, and no aBMD measures were studied. |

**Supplementary Table 2.** Summary of 52 pediatric DXA studies evaluating bone mineral density (BMD) by race/ethnicity. Findings varied by analytic approach: 12 reported no racial differences, 10 reported mixed or attenuated differences after covariate adjustment, 26 reported persistent differences, and 4 presented descriptive data only. The strength of reported differences depended largely on metric choice, covariate adjustment, and study design.

Footnote:

† studies that assessed BMD using absolute values (g/cm²).

‡ studies that assessed BMD using standardized BMD Z-scores

*studies that assessed bone mass

^a^ studies that assessed BMC (absolute values)
